# Intra-Articular Injection of Cross-Linked Hyaluronic Acid-Dexamethasone Hydrogel Attenuates Osteoarthritis: An Experimental Study in a Rat Model of Osteoarthritis

**DOI:** 10.3390/ijms17040411

**Published:** 2016-04-15

**Authors:** Zhiwei Zhang, Xiaochun Wei, Jizong Gao, Yu Zhao, Yamin Zhao, Li Guo, Chongwei Chen, Zhiqing Duan, Pengcui Li, Lei Wei

**Affiliations:** 1 Shanxi Key Laboratory of Bone and Soft Tissue Injury Repair, Department of Orthopedics, The Second Hospital of Shanxi Medical University, No. 382, Wuyi Road, Taiyuan 030001, China; weixiaochun11@126.com (X.W.); zhaoyu20806@163.com (Y.Z.); guol0918@126.com (L.G.); chenchongwei.ty@163.com (C.C.); ncbivip@163.com (Z.D.); lpc1977@163.com (P.L.); 2 BioRegen Biomedical (Changzhou) Co. Ltd., 167-5 East East Road, Changzhou 213025, China; jzgao@bioregenmed.com (J.G.); ymzhao@bioregenmed.com (Y.Z.); 3 Department of Orthopedics, Warren Alpert Medical School of Brown University, Suite 402A, 1 Hoppin Street, Providence, RI 02903, USA

**Keywords:** cross-linked hyaluronic acid, dexamethasone, intra-articular injection, osteoarthritis

## Abstract

Cross-linked hyaluronic acid hydrogel (cHA gel) and dexamethasone (Dex) have been used to treat knee osteoarthritis (OA) in clinical practice owing to their chondroprotective and anti-inflammatory effects, respectively. The aim of the present study was to compare the treatment effects of the cHA gel pre-mixed with/without Dex in a surgery-induced osteoarthritis model in rats. Anterior cruciate ligament transection (ACLT) surgery was performed on the right knee of rats to induce OA. Male 2-month-old Sprague-Dawley rats were randomly divided into five groups (*n* = 10/per group): (1) ACLT + saline; (2) ACLT + cHA gel; (3) ACLT + cHA-Dex (0.2 mg/mL) gel; (4) ACLT + cHA-Dex (0.5 mg/mL) gel; (5) Sham + saline. Intra-joint injections were performed four weeks after ACLT in the right knee. All animals were euthanized at 12 weeks post-surgery. Cartilage damage and changes in the synovial membrane were assessed by micro X-ray, Indian ink articular surface staining, Safranin-O/Fast Green staining, immunohistochemistry, hematoxylin and eosin staining of the synovial membrane, and quantitative reverse transcription-polymerase chain reaction for changes in gene expression. Micro X-ray revealed that the knee joint treated with the cHA-Dex gel was wider than those treated with cHA gel alone or saline. The cHA-Dex gel group had less Indian ink staining (indicator of cartilage fibrillation) than the cHA gel or saline injection groups. Safranin-O/Fast Green staining indicated that increased proteoglycan staining and less cartilage damage were found in the cHA-Dex gel group compared with the cHA gel or saline injection groups. Quantification of histology findings from saline, cHA gel, cHA-Dex (0.2 mg/mL) gel, cHA-Dex (0.5 mg/mL) gel, and sham groups were 5.84 ± 0.29, 4.50 ± 0.87, 3.00 ± 1.00, 2.00 ± 0.48, and 0.30 ± 0.58 (*p* < 0.05), respectively. A strong staining of type II collagen was found in both the cHA-Dex gel groups compared with saline group or cHA alone group. Similar result was found for the mRNA level of aggrecan and opposite result for type X collagen. Hematoxylin and eosin staining in the synovial membrane showed less synovial lining cell layers and reduced inflammatory cell infiltration in cHA-Dex gel-treated animals compared with saline or cHA only groups. Altogether, cHA-Dex gel has better chondroprotective and anti-inflammatory effects in rat surgery-induced osteoarthritis than cHA alone.

## 1. Introduction

Osteoarthritis (OA) is an important cause of chronic pain and disability worldwide among the elderly population [1]. Current pharmacological, non-pharmacological, and surgical treatments for OA can alleviate some symptoms, but do not attenuate disease progression [2]. Drugs, such as glucocorticoids and non-steroidal anti-inflammatory drugs (NSAIDs), have been widely used for the treatment of OA; however, none of the current drugs can prevent cartilage degeneration or completely cure this intractable disease. Furthermore, many of the drugs are not ideal for long-term treatment because of some adverse effects [3]. The development of disease-modifying osteoarthritis drugs (DMOADs) has been the focus of many current therapeutics of OA [4,5,6].

Corticosteroids have been used to treat OA for many years and rheumatologists have substantial clinical experience of their efficacy. Dexamethasone (Dex), an anti-inflammatory agent, has been widely recommended as an important complementary treatment for knee OA [7,8,9]. Recent clinical trials and a meta-analysis have also demonstrated the effectiveness of corticosteroids [10]. The protective effects of Dex on cartilage degeneration have been observed in animal experiments and clinical studies [11,12]. In addition, Dex is a key reagent for inducing chondrogenesis of mesenchymal stem cells (MSCs) *in vitro* [13]. These benefits of Dex are some of the ideal properties of DMOADs, which has promoted the wide use of Dex in OA treatment, despite its complications.

Intra-articular injection of hyaluronic acid (HA) to treat OA has been used worldwide for pain relief and symptomatic treatment [14,15]. The two types of HA currently available are low molecular weight (LMW) HA (molecular weight 0.5–3.6 million Da) and high molecular weight (HMW) chemically cross-linked HA (cHA, molecular weight 6.0 million Da) [16]. It has been reported that non-modified LMW HA has a half-life of only 10–13 h, while chemically-modified HA products, such as hylan G-F 20, can last for 8.8 ± 0.9 days [17,18]. The rapid clearance and elimination of LMW HA from the intra-articular cavity may have contributed to inefficient results in some patients. cHA with longer residence time has been used as second generation HA for intra-articular injection.

A recent meta-analysis comparing the therapeutic effect of intra-articular injection of HA with that of corticosteroids for knee OA (seven trials; *n* = 583 patients) showed that corticosteroids and HA exhibited equal efficacy in the short term (one month), but that HA had an enhanced effect in the long term (six months) [19]. Thus, the combination of these two agents may bring about a synergistic effect in the treatment of OA. In addition, the slow release of Dex from cHA may sustain its therapeutic effect over the long term. Therefore, we produced an injectable cHA-Dex gel and hypothesized that the combination of cHA and Dex would produce a synergistic effect in OA treatment, through their chondrogenic and anti-inflammatory effects, respectively. This combination could be an ideal DMOAD.

## 2. Results

### 2.1. Cross-Linked Hyaluronic Acid-Dexamethasone (cHA-Dex) Hydrogel Exhibited Low Cytotoxicity in Vitro Compared with Dex Alone

**Figure 1:**
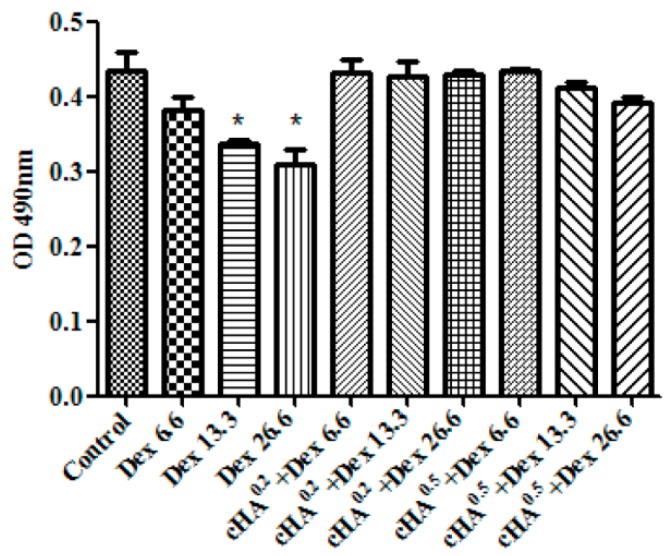
Cross-linked hyaluronic acid-dexamethasone (cHA-Dex) hydrogel exhibited low cytotoxicity compared with Dex only treatment *in vitro*. The influence of Dex, cHA-Dex (0.2 mg/mL) gel, and cHA-Dex (0.5 mg/mL) gel on the survival of human osteoarthritic chondrocytes was assessed using a methyl thiazolyl tetrazolium (MTT)-based colorimetric assay. Three Dex concentrations were tested, which were 6.6, 13.3, and 26.6 µg/mL. Data were expressed as means ± SD. * represents that the difference was statistically significant (* *p* < 0.05). *n* = 3.

As shown in Figure 1, the viability of human OA chondrocytes was noticeably influenced by 13.3 and 26.6 µg/mL Dex (*p* < 0.05). The negative influence of Dex on cell viability was significantly reduced when Dex was mixed in the cHA gel that contained different concentrations of cHA. Similar results were also observed *in vitro* where the growth of the human OA chondrocytes incubated with cHA-Dex (0.2 mg/mL) gel or cHA-Dex (0.5 mg/mL) gel were better than those treated with Dex alone (Supplementary Materials Figure S1).

### 2.2. Intra-Articular cHA-Dex Hydrogel Injection Attenuated Osteoarthritis (OA) Macroscopically

**Figure 2:**
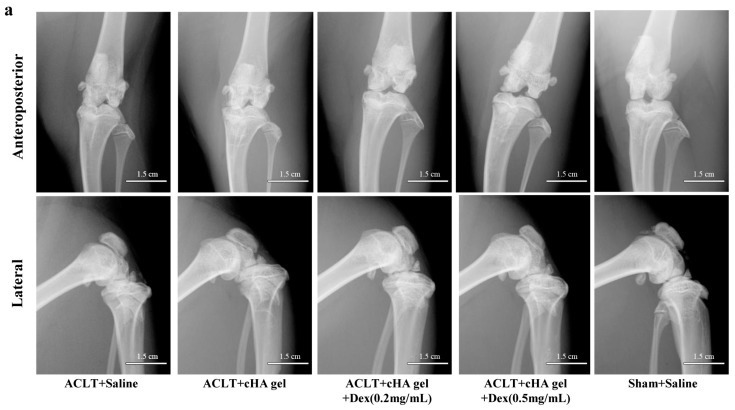

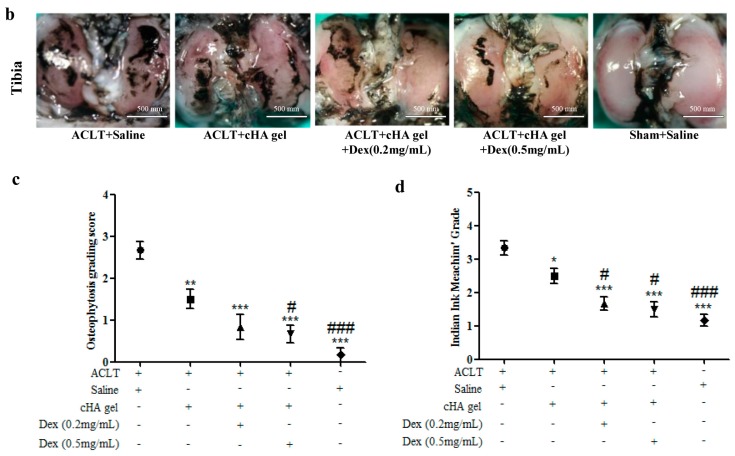
Intra-articular cross-linked hyaluronic acid-dexamethasone (cHA-Dex) hydrogel injection attenuated osteoarthritis (OA) macroscopically. Following anterior cruciate ligament transection (ACLT) surgical induction of OA, micro X-Ray (**a**) was used to evaluate the severity of OA in the right knee before the animals were euthanized; (**b**) Gross morphological cartilage lesion and fibrillation in the rat tibial plateau were visualized by Indian ink staining; (**c**) Radiographic osteophytosis was graded subjectively on a scale from 0 to 3 (0-normal, 1-mild, 2-moderate, 3-severe) based on the severity of osteophytosis at the margins of the knee joint; (**d**) Cartilage lesions and fibrillation was quantified using the Meachim grading system. Data were expressed as means ± SD. *, compared with ACLT + saline group, designated as * *p* < 0.05, ** *p* < 0.01 and *** *p* < 0.001; #, compared with ACLT + cHA gel group, designated as # *p* < 0.05, and ### *p* < 0.001.*n* = 10.

At 12 weeks after anterior cruciate ligament transection (ACLT), the knee joint space was significantly wider in the cHA-Dex (0.2 mg/mL) gel and cHA-Dex (0.5 mg/mL) gel groups, compared with the saline or cHA only groups (Figure 2a), indicating that intra-articular injection of the cHA-Dex gel exhibited preventative effects on OA. Radiographic osteophytosis grading system, which quantifies the severity of osteophytosis at the margins of the knee joint, showed that osteophytosis in the cHA gel only, cHA-Dex (0.2 mg/mL) gel and cHA-Dex (0.5 mg/mL) gel groups were significantly lower than that of saline group (*p* < 0.01). In addition, less osteophytes were found in the cHA-Dex (0.5 mg/mL) gel group compared with the cHA gel only group (*p* < 0.05) (Figure 2c).

Gross morphological cartilage lesion and fibrillation in the rat tibial plateau (*n* = 10/group) were visualized by Indian ink staining (Figure 2b). The Indian ink staining Meachim grading system indicated that the cHA-Dex gel group had less cartilage lesion and fibrillation than the cHA gel only, or saline injection groups, but more than in the sham control group (*p* < 0.05) (Figure 2d).

### 2.3. Intra-Articular cHA-Dex Hydrogel Injections Increased the Expression of Proteoglycan in the Microscopic Appearance of OA Pathology

**Figure 3:**
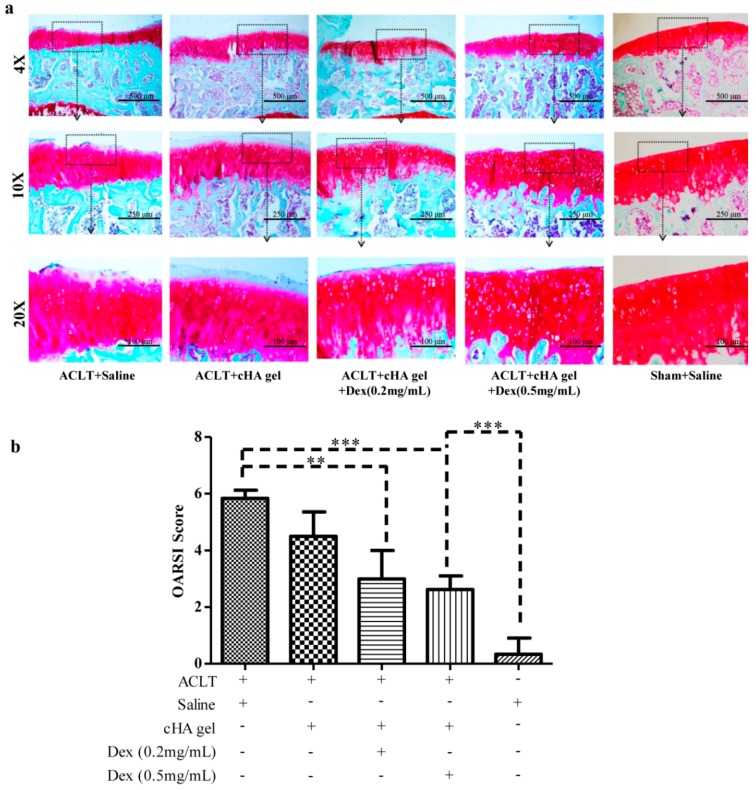
Intra-articular cross-linked hyaluronic acid-dexamethasone (cHA-Dex) hydrogel injections increased the expression of proteoglycan in the microscopic appearance of osteoarthritis (OA) pathology. 6 µm thick histological sections of cartilage from the tibial plateau were stained with Safranin-O/Fast Green. A smoother surface and less blue fibrosis (**a**) was detected in the articular cartilage of the cHA-Dex gel injection animals comparing with the ACLT + saline and cHA only group; (**b**) OARSI histological grading score (mean ± SD) indicated that cartilage damage in the ACLT + saline group was the most severe of all the groups, while cartilage in the sham+saline group had the least damage. Cartilage damage was less in the cHA-Dex (0.2 mg/mL) and cHA-Dex (0.5 mg/mL) gel groups than in the ACLT + saline group. * represents that the difference was statistically significant, ** *p* < 0.01 and *** *p* < 0.001.*n* = 10.

Histological examination showed that treatment with either the cHA-Dex gel or the cHA gel attenuated cartilage lesion formation, whereas stronger Safranin O staining, with more cellularity and less losses of the superficial layer and less fibrosis were observed in the cHA-Dex gel group. The cartilage in the cHA-Dex (0.5 mg/mL) gel group had stronger proteoglycan staining and a more intact surface than the cHA-Dex (0.2 mg/mL) gel group, but was weaker than that of the sham control group (Figure 3a). The Osteoarthritis Research Society International (OARSI) cartilage histological grading scores (Figure 3b) in both cHA-Dex gel groups suggested mild degeneration (3.0 ± 1.0 and 2.0 ± 0.48, respectively. *p* < 0.01), while cartilage damage in the cHA gel and saline groups were significantly more severe (4.5 ± 0.87 and 5.84 ± 0.29, respectively. *p* < 0.01). The cartilage in the sham group had the lowest severity of damage (0.3 ± 0.58, *p* < 0.001). Histologic changes were only evaluated at 12 weeks.

### 2.4. Intra-Articular cHA-Dex Hydrogel Injections Increased COL II Expression, but Decreased COL X and MMP-13 Expression in Cartilage, Histologically

**Figure 4:**
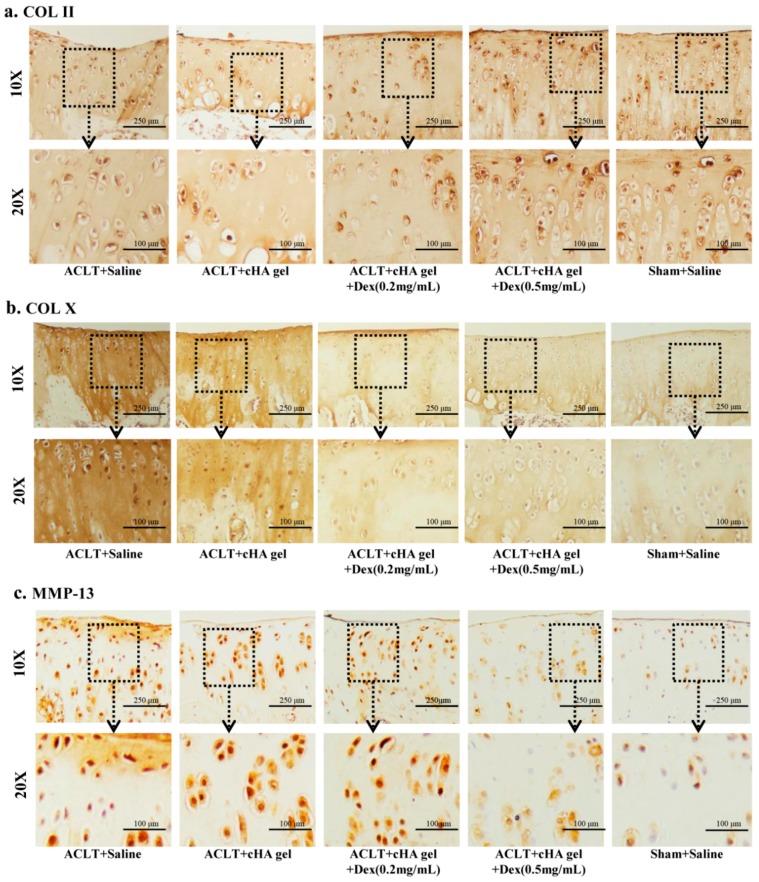
Intra-articular cross-linked hyaluronic acid-dexamethasone (cHA-Dex) hydrogel injections increased type II collagen (COL II) expression, while it decreased type X collagen (Col X) and matrix metalloproteinase 13 (MMP-13) expressions in cartilage, histologically. Immunohistochemical staining was used for COL II, COL X, and MMP-13. Positive signals are indicated by brown staining. Increased COL II expression (**a**), and decreased COL X (**b**) and MMP-13 (**c**) expressions were detected in the articular cartilage of cHA-Dex gel-treated animals comparing with the ACLT + saline or cHA only groups. The images obtained under the 10×, 20× objective lenses respectively.

Immunohistochemistry revealed that COL II staining in both cHA-Dex gel-treated groups was stronger than that in the cHA gel or saline groups (Figure 4a), and showed Dex-dose dependency. COL X and MMP-13 were weakly expressed in the cartilage of both cHA-Dex gel-treated groups, but it was more extensively visible in the cHA gel only group, and even more so in the saline group (Figure 4b,c). In addition, there was less immunostaining for COL X and MMP-13 in the cHA-Dex (0.5 mg/mL) gel-treated animals compared with the cHA-Dex (0.2 mg/mL) gel-treated animals.

### 2.5. Intra-Articular cHA-Dex Hydrogel Injections Relieved Synovial Inflammation

**Figure 5:**
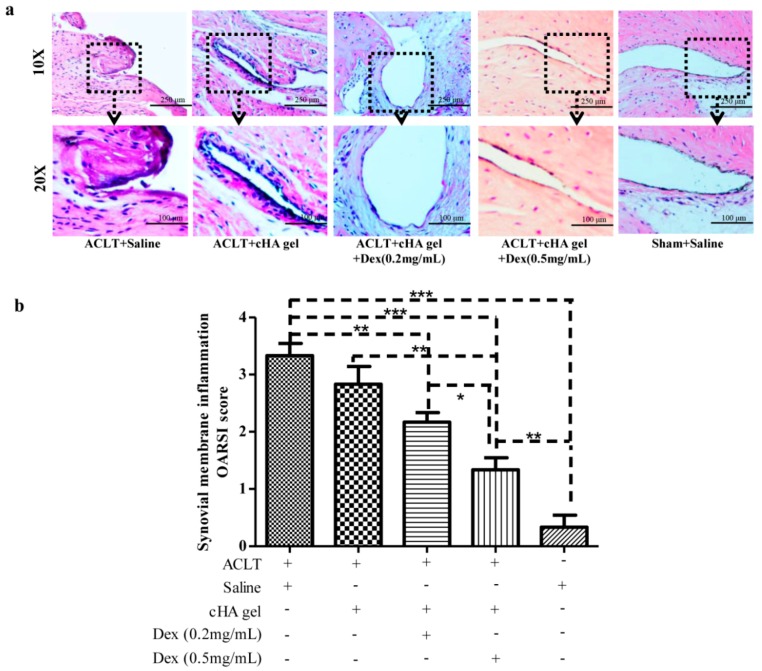
Intra-articular cross-linked hyaluronic acid-dexamethasone (cHA-Dex) hydrogel injections relieved synovial inflammation. Hematoxylin and eosin staining (**a**) showed lower numbers of synovial lining cell layers and reduced infiltration of inflammatory cells in the synovial membrane of cHA-Dex gel-treated animals comparing with the ACLT + saline or cHA only groups; (**b**) Synovial membrane inflammation OARSI score indicated that the inflammatory response of cHA-Dex (0.5 mg/mL) gel group was alleviated compared with that in the cHA gel only or ACLT + saline control groups. Data were expressed as means ± SD. * represents that the difference was statistically significant, * *p* < 0.05, ** *p* < 0.01 and *** *p* < 0.001. *n* = 10.

The sham group showed normal synovial tissue at 12 weeks post-surgery (Figure 5a). The synovial surface of both cHA-Dex gel-treated groups had lower numbers of synovial lining cell layers and exhibited infiltration of inflammatory cells similar to that of the sham group. Furthermore, the inflammatory response of the cHA gel only group was alleviated compared with that of the saline groups, but was no better than that of both cHA-Dex gel-treated groups, especially the cHA-Dex (0.5 mg/mL) gel-treated group.

Synovial membrane inflammation OARSI score in the right knee joint (Figure 5b) demonstrated a milder inflammatory response in the synovial membrane of the cHA-Dex (0.5 mg/mL) gel-treated animals comparing with the cHA gel alone group (*p* < 0.01). No difference in inflammation was found between the cHA gel only and saline control groups. The cHA gel group had greater synovial lining layers and inflammatory cells, but the differences were not statistically significant compared with the cHA-Dex (0.2 mg/mL) gel group.

### 2.6. Intra-Articular cHA-Dex Hydrogel Injections Increased Gene Expression of Aggrecan, but Decreased Type X Collagen

**Figure 6:**
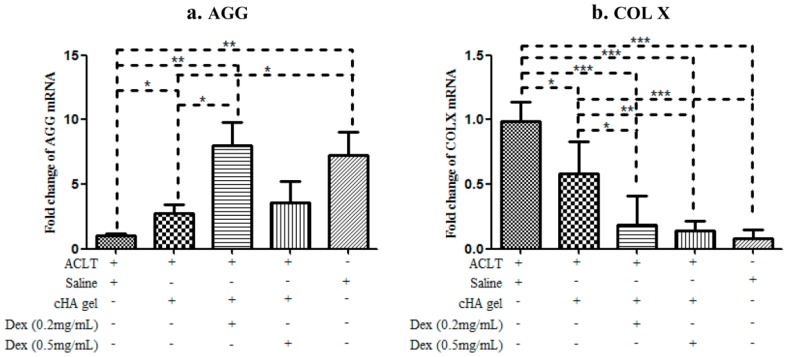
Intra-articular cross-linked hyaluronic acid-dexamethasone (cHA-Dex) hydrogel injections increased aggrecan (AGG) and decreased type X collagen (COL X) gene expression. (**a**) The mRNA level of AGG was increased in the cHA-Dex (0.2 mg/mL) gel group compared with the ACLT + saline or cHA only groups (*p* < 0.05), suggesting a positive impact of the cHA-Dex gel on anabolic metabolism. (**b**) In contrast, COL X was decreased compared with the ACLT + saline or cHA only groups (*p* < 0.05). Data were expressed as means ± SD. * represents that the difference was statistically significant, * *p* < 0.05, ** *p* < 0.01 and *** *p* < 0.001. *n* = 10.

RT-qPCR results indicated that intra-articular cHA-Dex gel injections enhanced the levels of aggrecan (AGG) mRNA, and suppressed those of COL X (Figure 6). COL X mRNA level in the saline or cHA gel only groups was significantly higher than that in the cHA-Dex (0.2 mg/mL) gel, cHA-Dex (0.5 mg/mL) gel and sham groups (*p* < 0.001). The mRNA level of COL X in saline group was highest among the five groups. In contrast, the AGG mRNA level in the cHA-Dex (0.2 mg/mL) gel or sham groups was significantly higher than that in the saline or cHA gel only group (*p* < 0.05). These data suggested that the cHA-Dex gel had a chondroprotective effect *in vivo* by decreasing the gene expressions of catabolic factors and hypertrophic marker, while increasing those of anabolic factors.

## 3. Discussion

Our *in vitro* data showed that treatment with 13.3 or 26.6 µg/mL Dex alone was toxic to human OA chondrocytes. However, the HA-Dex gel displayed significantly lower cytotoxicity, compared with similar concentrations of Dex alone. These results suggest that the combination of a cHA gel with Dex significantly reduced the cytotoxicity caused by Dex alone. The cHA gel may have reduced the peak concentration of Dex by slowly releasing the drug, thereby avoiding high concentrations of Dex in the culture medium. Further research is being performed to investigate the release profile of Dex from the cHA gel.

Micro X-ray and Indian ink staining revealed that both the cHA gel and the cHA-Dex gel intra-articular injections attenuated rat OA compared with the saline group. However, the cHA-Dex gel treatments exhibited greater relief from OA than the cHA gel only group. Histology and RT-qPCR data showed similar results. Both the cHA-Dex (0.2 mg/mL) gel and cHA-Dex (0.5 mg/mL) gel treatments reduced cartilage degradation. In Figure 6a, we noticed that aggrecan mRNA in cHA-Dex (0.5 mg/mL) is lower than that in cHA-Dex (0.2 mg/mL). However, in all other analyses, cHA-Dex (0.5 mg/mL) has same or higher effect than cHA-Dex (0.2 mg/mL). This discrepancy may be caused by epigenetic inheritance or variation of aggrecan and regulation of some unknown reasons after treatment with cHA-Dex in our study. We also noticed that the higher level mRNA of aggrecan in the cHA-Dex-treated animals compared with cHA treated animals alone. The result could be a combination effect from cHA-Dex chondroprotection with more aggrecan production, because the chondrocytes may try to produce more aggrecan to balance the loss of aggrecan in the OA early stage. In addition, the cHA-Dex (0.5 mg/mL) gel group also had a reduced inflammatory response in the synovial membrane compared with the cHA gel only, or saline control groups. All of the data collectively supports the cHA-Dex gel treatments exhibited chondroprotective and antiphlogistic effects, which were superior to those of the cHA gel only treatment.

HA is a primary component in synovial fluid and articular cartilage matrix. The maintenance of normal joint function is tightly correlated with a normal concentration of HA in the synovial fluid [20]. Intra-articular injection of HA can cover the articular surfaces and improve nutrient transport in cartilage, acting as a cushion that absorbs pressure and friction, thus protecting it from further damage. The adherence of HA to knee articular cartilage surfaces has been reported to protect nerve endings that may be exposed by cartilage degradation [21,22]. Intra-articular treatment with HA for OA knee pain is widely accepted in clinical practice [23]. This method can supply sufficient HA to delay the development of knee OA [24,25]. Our study indicated that cHA-Dex gel injections might improve repair and protection against early OA by delaying cartilage destruction via inhibition of MMP-13 expression, which was confirmed by our histological findings. Since MMP-13 has a potent ability to degrade the proteoglycan of articular cartilage, it is recognized as an enzyme that plays a critical role in the degeneration of articular cartilage in OA. The results of this study demonstrated that MMP-13 expression was elevated and sustained in the control group treated with saline, whereas MMP-13 levels were significantly decreased in the cHA-Dex gel treatment groups compared with the cHA only group. Thus, the cHA-Dex gel may prevent MMP-13 secretion, which in turn rescues COL II and proteoglycan expression.

Several different HA-based combination compounds were recently reported to have therapeutic effects following intra-articular injection in an animal OA model. HA-celecoxib gel was more effective than a single drug in achieving pain relief and articular cartilage protection [26]. Intra-articular injection of HA-doxycycline was more effective for pain control and in delaying cartilage degeneration by preventing proteoglycan loss [27]. Collagen tripeptide and HA combination was also reported to be effective in preventing early articular cartilage degeneration by inducing increased COL II expression. An important goal of HA intra-articular injection is to restore the normal viscoelasticity and natural protective functions of the synovial fluid in the knee joint. It has been reported that the molecular weight (*M*w) of HA in a healthy knee joint is 6 × 10^6^ Da in humans, while the *M*w of HA decreases to 0.5–3.0 × 10^6^ Da in the osteoarthritic knee joint [28]. cHA is cross-linked and could have theoretically large *M*w. This cHA is capable to remaining in the joint for a much longer length of time than natural HA, thus providing sustained improvements in joint lubrication and an enhanced chondroprotective effect [29]. This advantage of cHA was enhanced by mixing with Dex, because Dex is able to inhibit the inflammatory reaction and reduce the production of cytokines. Mixing of Dex inside the cHA gel allowed its slow release into the joint cavity, where it had a therapeutic role.

This study still has several limitations. Firstly, although rats were chosen in our study, they are not upright walking animals. To address this limitation, sheep OA models have been used in another preclinical experiment to further investigate the effect of cHA-Dex gel on OA. Secondly, the treatment was started at 4 weeks post-surgery in our study, which may limit the cHA-Dex gel therapeutic outcomes. It has been reported that similar HMW cHA-hylan G-F 20 injections before 4 weeks post-surgery had protective effects in delaying cartilage degeneration and decreasing the formation of osteophytes in an OA model [30]. This suggests the potential early application of cHA-Dex gel can be seen from close to the onset of OA. Future studies with different therapeutic regimens of cHA-Dex gel could provide additional support to our current findings. Finally, many researchers believe that the pathological changes of OA not only include articular cartilage degeneration, but also cause changes in the subchondral bone, joint capsule and other joint tissue. In our ongoing sheep experiment, the subchondral bone and anti-inflammatory effects of intra-articular cHA-Dex gel treatment in OA are being investigated further.

## 4. Materials and Methods

### 4.1. Cell Viability and Toxicity

To evaluate the influence of cHA with or without Dex, human OA chondrocytes, obtained from osteoarthritic patients undergoing total knee arthroplasty, were cultured in the presence of Dex, cHA gel, and cHA-Dex gel (provided by BioRegen Biomedical Inc, Changzhou, China). Human chondrocytes were isolated as previously described [31] and plated in 96-well culture plates at a density of 1 × 10^6^ cells/plate. Three Dex concentrations (6.6, 13.3, and 26.6 µg/mL) were tested. The survival of human OA chondrocytes was assessed using methyl thiazolyl tetrazolium (MTT) assay, according to the standard protocol, 24 h after treatments. The study was approved by the Institutional Review Board at Shanxi Medical University (approval project identification code: 2013025, date: January 2013 to December 2018), and informed consent was obtained from each tissue donor.

### 4.2. Rat Anterior Cruciate Ligament Transection (ACLT) OA Model and Treatment with Intra-Articular cHA-Dex Hydrogel Injections

To investigate the treatment effects of cHA gel or cHA gel with Dex *in vivo*, we used an animal model of OA in Sprague–Dawley (SD) rats, which were acquired from Shanxi Medical University Experimental Animal Center and approved by the Shanxi Medical University Institutional Animal Care and Use Committee. A total of fifty two-month-old SD rats (weighing 180–230 g) were randomly divided into five groups (*n* = 10 per group): (1) anterior cruciate ligament transection (ACLT) + saline; (2) ACLT + cHA gel; (3) ACLT + cHA-Dex (0.2 mg/mL) gel; (4) ACLT + cHA-Dex (0.5 mg/mL) gel; and (5) sham + saline. The cHA gel and cHA-Dex gel were premixed and prefilled in glass syringes that were provided sterile after autoclaving. ACLT or sham surgeries were performed on the right knees, by the same methods published previously [32]. Intra-articular injections (50 µL) were performed four weeks after ACLT, with each animal receiving only one injection. Animals in groups 1 and 5 received an equivalent volume of saline injection into their right knees at the same time points as the experimental groups 2, 3, and 4 to avoid any procedural effects. All animals were euthanized at week 12 after the ACLT surgery. Tibia plateaus were used for histology and femoral condyles were used for gene analysis by quantitative reverse transcription polymerase chain reaction (RT-qPCR).

### 4.3. Radiography

The knee joints were examined with micro X-ray (Faxitron Bioptics, Lincolnshire, IL, USA) to evaluate the severity of OA before the animals were euthanized. Radiographic grading was based on previously published numerical rating scales [33]. Osteophytosis was graded subjectively on a scale from 0 to 3 (0-normal, 1-mild, 2-moderate, 3-severe) based on its severity at the margins of the knee joint. Radiographic scoring was performed by a single surveyor (Zhiwei Zhang), after training was provided by an experienced surgeon (Xiaochun Wei).

### 4.4. Histology

After the knee joints were opened and disarticulated, the gross morphological lesions on the rat tibial plateaus (*n* = 10/group) were visualized by Indian ink staining. Cartilage lesions and fibrillation were quantified using the Meachim grading system [34]. After Indian ink staining, the tibial plateaus were fixed in 10% formalin for three days and decalcified in EDTA solution. The specimens were hemi-sectioned in the mid-sagittal plane, then each half was embedded in a single block of Paraplast X-tra (Fisher, Santa Clara, CA, USA). Blocks were trimmed to expose the articular cartilage. Ten adjacent sections were collected at intervals of 0, 100, and 200 µm. Ten serial 6-μm thick sections from each interval were stained with Safranin-O/Fast Green. Cartilage degradation was quantified using the OARSI cartilage grading system [35]. Three independent observers scored each section, and the scores for all of the sections cut from the medial and lateral tibial plateaus were averaged within each joint. All scorings were performed by three independent observers who were not aware of the treatment procedures received by each joint.

### 4.5. Immunohistochemistry

Immunohistochemistry was performed on all specimens to detect type II collagen (COL II), type X collagen (COL X), and matrix metalloproteinase 13 (MMP-13) using the Histostain-SP Kit (Zymed-Invitrogen, Carlsbad, CA, USA). The sections were digested with 5 mg/mL of hyaluronidase (Sigma-Aldrich, St Louis, MO, USA) for 20 min in 37 °C. Normal serum blocking solution (LICOR, Lincoln, NE, USA) was used to prevent nonspecific binding. The sections were incubated with primary antibody against rat COL II (2 µg/mL) (Santa Cruz, CA, USA), COL X (2 µg/mL) (Santa Cruz, CA, USA) and MMP-13 (2 µg/mL) (Santa Cruz, CA, USA) at 4 °C overnight. Thereafter, the sections were treated sequentially with biotinylated secondary antibody and streptavidin-peroxidase conjugate (Zymed-Invitrogen), followed by standardized development in 3’3-diaminobenzidine (DAB). Images were obtained with a Nikon E800 microscope (Nikon, Melville, NY, USA).

### 4.6. Evaluation of Synovitis

Separate joint sections were stained with hematoxylin and eosin (H and E) to observe the inflammatory response in the synovial membrane. The numbers of synovial lining cell layers, proliferation of subsynovial tissue, and infiltration of inflammatory cells in the synovium were scored using the synovial membrane inflammation OARSI grade system [35].

### 4.7. Quantitative Reverse Transcription Polymerase Chain Reaction (RT-qPCR)

Cartilage tissue was collected from the femoral condyle of each knee joint and ground using a mortar and pestle with some liquid nitrogen. Total RNA was isolated from rat knee articular cartilage using Trizol reagent (Invitrogen, Carlsbad, CA, USA). Cartilage samples from two rat femur condyles were dissected with a scalpel and pooled together. Five pooled samples per group were used for RT-qPCR (*n* = 10/group). One µg of total RNA was reversed transcribed to cDNA with the iScriptTM cDNA synthesis Kit (Fermentas, Glen Burnie, MD, USA). Forty ng/µL of the resulting cDNA was used as the template to perform real-time qPCR amplification using the QuantiTect SYBR Green PCR Kit (Fermentas) with IQ 5.0 (Bio-Rad, Hercules, CA, USA). Rat aggrecan (AGG) forward primer—5’-CAGTGCGATGCAGGCTGGCT-3’, reverse primer—5’-CCTCCGGCACTCGTTGGCTG-3’; Rat Col10a1 (Type X collagen) forward primer—5’-CCAGGTGTCCCAGGATTCCC-3’, reverse primer—5’-CAAGCGGCATCCCAGAAAGC-3’; 18S RNA forward primer—5’-CGGCTACCACAT CCAAGGAA-3’, reverse primer—5’-GCTGGAATTACCGCGGCT-3’. 18S was used as an internal control gene to normalize the mRNA levels. Amplification conditions were as follows: 2 min of pre-incubation at 50 °C, 10 min at 95 °C for enzyme activation, and 40 cycles of denaturation at 95 °C for 10 s, annealing at 55 °C for 30 s, and extension at 72 °C for 30 s. Relative transcription levels were calculated by *x* = 2^−ΔΔ*C*t^ [36].

### 4.8. Statistical Analysis

SPSS 17.0 (SPSS Inc., Chicago, IL, USA) was used for the statistical analysis. All data in this study were expressed as mean ± standard deviation (SD). One-way analysis of variance (ANOVA) and Tukey tests were used to compare the data in different groups. A *p* value of less than 0.05 was considered statistically significant.

## 5. Conclusions

This is the first report that cHA-Dex hydrogel has a better chondroprotective and anti-inflammatory effects in rat OA models than cHA treatment alone and that it can attenuate cartilage damage.

## Supplementary Materials

Supplementary materials can be found at http://www.mdpi.com/1422-0067/17/4/411/s1.
