# Supplementary material for: Intra-Articular Injection of Cross-Linked Hyaluronic Acid-Dexamethasone Hydrogel Attenuates Osteoarthritis: An Experimental Study in a Rat Model of Osteoarthritis
Source: Int J Mol Sci. 2016 Apr 15;17(4):411. doi: 10.3390/ijms17040411 (PMC4848885; doi:10.3390/ijms17040411)
Supplement: Supplementary file 1 [file ijms-17-00411-s001.pdf]

Supplementary Materials: Intra-Articular Injection of Cross-Linked Hyaluronic Acid-Dexamethasone Hydrogel Attenuates Osteoarthritis: An Experimental Study in a Rat Model of Osteoarthritis

Zhiwei Zhang, Xiaochun Wei, Jizong Gao, Yu Zhao, Yamin Zhao, Li Guo, Chongwei Chen, Zhiqing Duan, Pengcui Li and Lei Wei

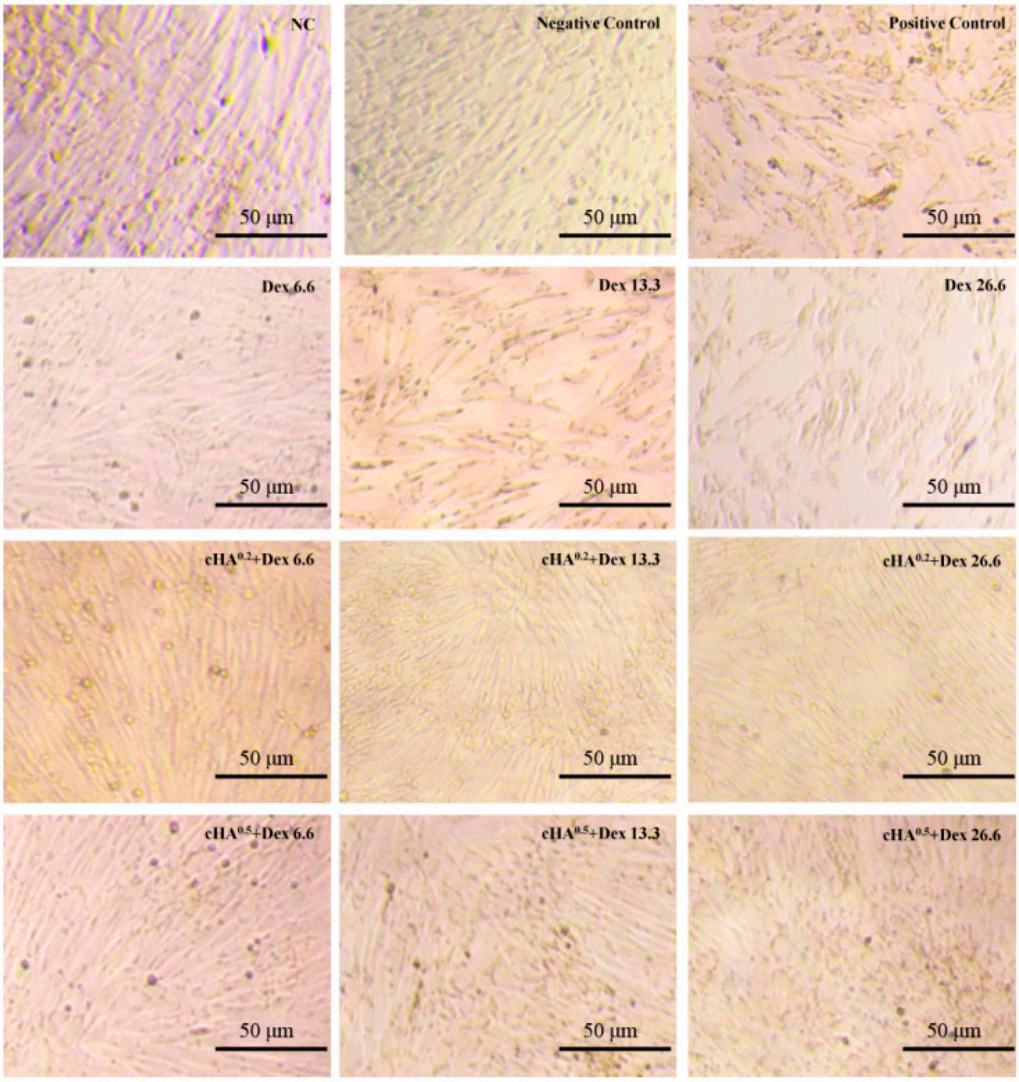

Figure S1. cHA-Dex hydrogel exhibited low cytotoxicity *in vitro* compared with Dex alone.
